# Integrative insights into abiotic stress tolerance in finger millet (*Eleusine coracana* (L.) Gaertn.): linking physiological, biochemical, and molecular perspectives for developing climate-smart cereals

**DOI:** 10.3389/fpls.2026.1815325

**Published:** 2026-05-18

**Authors:** Kasinathan Rakkammal, Pandiyan Muthuramalingam, Subramani Pandian, Md Atikur Rahman, Md. Mezanur Rahman, S. M. Shahinul Islam, Theivanayagam Maharajan, Collince Omondi Awere, Stanislaus Antony Ceasar, Hyunsuk Shin, Manikandan Ramesh

**Affiliations:** 1 Department of Biotechnology, Alagappa University, Karaikudi, Tamil Nadu, India; 2 Department of GreenBio Science, College of Agriculture and Life Sciences, Gyeongsang National University, Jinju, Republic of Korea; 3 Department of Agricultural Biotechnology, National Institute of Agricultural Sciences, Rural Development Administration, Jeonju, Republic of Korea; 4 Institute of Biological Sciences, University of Rajshahi, Rajshahi, Bangladesh; 5 Institute of Genomics for Crop Abiotic Stress Tolerance, Department of Plant and Soil Science, Texas Tech University, Lubbock, TX, United States; 6 Biosciences Division, Oak Ridge National Laboratory, Oak Ridge, TN, United States; 7 Division of Plant Molecular Biology and Biotechnology, Department of Biosciences, Rajagiri College of Social Sciences, Cochin, Kerala, India

**Keywords:** abiotic stress tolerance, antioxidant enzyme, finger millet, genome editing, multi-omics

## Abstract

Finger millet (*Eleusine coracana* (L.) Gaertn.) is a resilient yet underutilized cereal with exceptional potential to withstand adverse environmental conditions. Despite its adaptability, the genetic and physiological bases of its stress tolerance remain insufficiently characterized and poorly integrated into modern crop improvement pipelines. This review critically evaluates current knowledge of finger millet responses to major abiotic stresses such as drought, salinity, temperature extremes, and heavy metal toxicity with the aim of identifying key regulatory nodes and translational opportunities. At the physiological level, finger millet displays notable plasticity through modulation of root system architecture, transpiration efficiency, and leaf structural traits. Biochemically, it accumulates osmoprotectants such as proline, enhances antioxidant enzyme activities, and activates detoxification pathways to mitigate cellular damage. These responses are orchestrated by diverse molecular regulators, including transcription factors, signaling proteins, and stress-responsive genes. However, a clear framework connecting physiological traits to molecular regulation remains lacking. Recent advances in genomics, transcriptomics, proteomics, and metabolomics provide powerful tools for dissecting complex stress adaptation networks. Furthermore, CRISPR/Cas9-mediated genome editing offers new avenues for precise trait enhancement. Yet, the incorporation of these technologies into breeding programs is still limited, particularly regarding genotype–phenotype associations and evaluation under multi-stress field conditions. This review highlights the integration of physiological, biochemical, and molecular strategies as a roadmap for precision breeding in finger millet. By linking molecular insights to practical agronomic outcomes, we advocate repositioning finger millet as a model system for developing climate-resilient cereals.

## 1 Introduction

The foremost challenge in modern agriculture is to enhance climate adaptability while increasing crop productivity and ensuring food security, particularly as the global population is projected to exceed 9 billion by 2050 (Giordano et al., 2021). A substantial rise in crop production is imperative to meet future food demands. Unfortunately, multiple environmental stress factors like water deficit, high salinity, low or high temperatures, limited nutrient availability, and heavy metals continue to significantly constrain crop yields (Rahman et al., 2024). Abiotic stress impairs plant growth, development, physiological functions, and ultimately diminishes harvestable yield. One of the primary consequences of such stresses is the overproduction of reactive oxygen species (ROS), which induce oxidative damage, disrupt cellular integrity, and accelerate programmed cell death (Samanta et al., 2024). To counteract these effects, plants have evolved a repertoire of adaptive strategies encompassing both physiological modifications and molecular regulatory mechanisms (Hasanuzzaman et al., 2020). Therefore, developing stress-resilient crop traits is essential for sustainable agriculture and global food security (Rahman et al., 2024).

Finger millet is a promising cereal crop with significant potential to contribute to global food security and economic development. The small-seeded grass crop exhibits remarkable stress-resilience and the capacity to adapt to diverse climatic conditions. In response to environmental stress signals, finger millet enhances its biochemical and molecular activities by increasing the production of compatible solutes that stabilize proteins and maintain their cellular structure (Mundada et al., 2020). It also regulates cell turgor through osmotic adjustments and activate its antioxidant defense system to restore redox homeostasis (Mundada et al., 2020). Abiotic stress modulates the expression of various genes involved in antioxidant defense, osmoprotectant biosynthesis, membrane transport, and regulatory protein networks (Goyal et al., 2024). These gene-mediated alterations in physiological and biochemical processes contribute significantly to stress tolerance. In this review, we provide a comprehensive overview of the physiological, biochemical, and molecular responses of finger millet under various abiotic stresses. We also outline several potential strategies aimed at enhancing stress tolerance, with a particular focus on the roles of integrated multi-omics technologies and genome editing approaches in advancing our understanding of stress adaptation in this resilient crop.

## 2 Finger millet: a timeless grain for modern wellness

**Figure 1:**
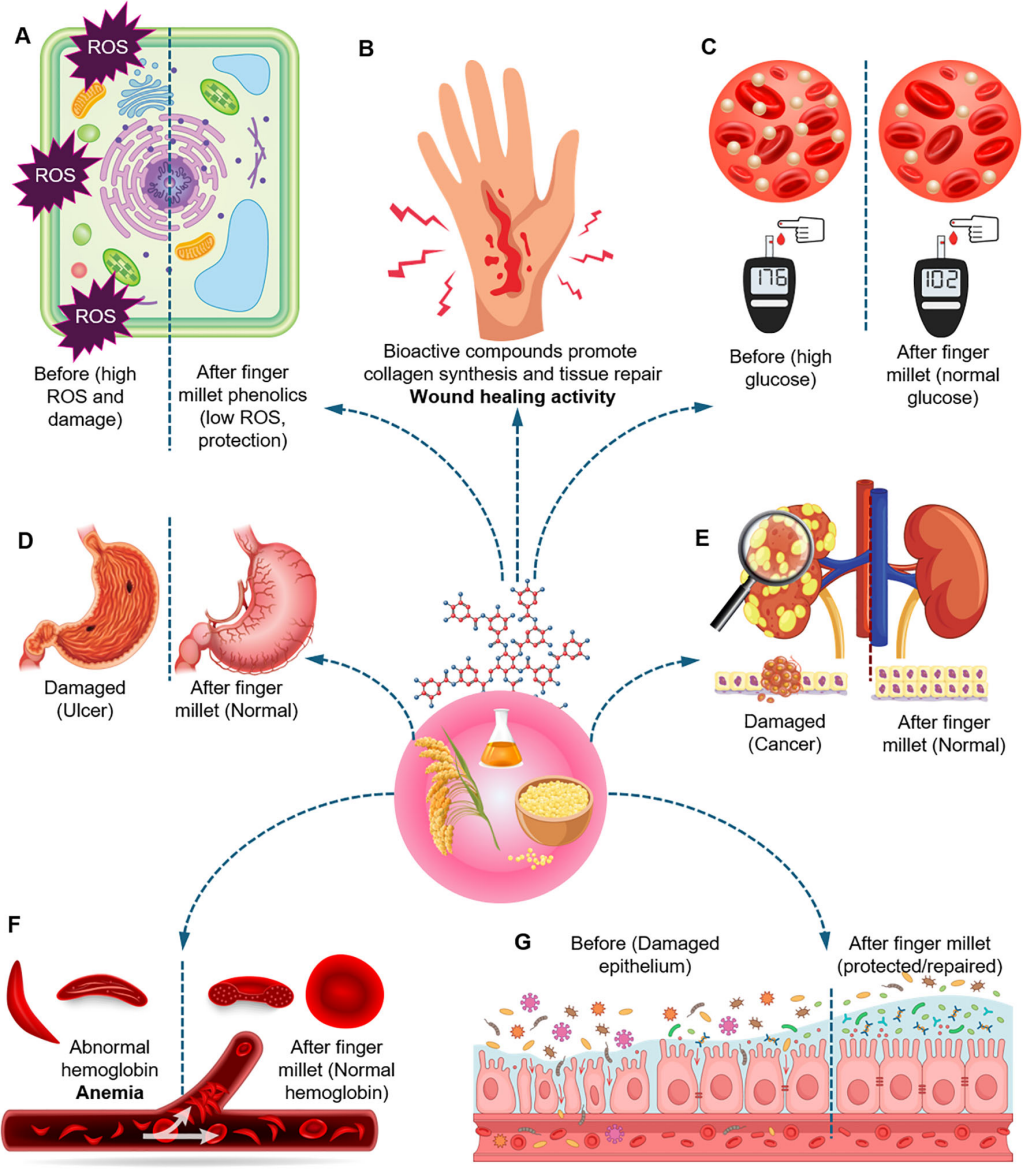
Proposed health−related activities of finger millet grain bioactives. **(A)** Cellular oxidative status before and after exposure to finger millet phenolics, illustrating reduced reactive oxygen species and protection from oxidative damage. **(B)** Wound surface showing stimulation of collagen synthesis and tissue repair, consistent with wound−healing activity. **(C)** Blood glucose profiles and circulating erythrocytes before and after finger millet intake, indicating improved glycemic control. **(D)** Gastric tissue before and after finger millet, highlighting protection of the mucosa consistent with antiulcer effects. **(E)** Renal and associated tissues depicting suppression of abnormal cell growth, consistent with anticancer potential. **(F)** Blood vessel and erythrocytes before and after finger millet, suggesting improved hemoglobin status and mitigation of anemia. **(G)** Intestinal or epithelial barrier before and after finger millet, showing restoration of epithelial integrity and reduced luminal insult, consistent with barrier protection and repair.

Finger millet, a C_4_ grass, is an important cereal crop cultivated primarily in arid and semi-arid regions. Its domestication is believed to have originated in Ethiopia and Uganda; since then, it has been widely cultivated across several Asian and African countries for both food and forage purposes (Vetriventhan et al., 2020). Globally, finger millet ranks as the fourth most widely grown millet, following foxtail millet (*Setaria italica*), pearl millet (*Cenchrus americanus*), and sorghum (*Sorghum bicolor*). In India, it is the sixth most common cereal crop after wheat (*Triticum aestivum*), rice (*Oryza sativa*), maize (*Zea mays*), sorghum, and pearl millet (Mbinda and Mukami, 2021; Sood et al., 2019). Finger millet is widely consumed across Asia and Africa in both traditional and modern dietary forms and is increasingly recognized for its substantial nutritional and health-promoting properties (Sood et al., 2019). It provides an excellent nutritional profile, encompassing dietary fiber, superior-quality proteins, and amino acids, as well as essential minerals such as calcium, iron, and magnesium, together with a diverse range of B-complex vitamins like thiamine, pyridoxine, niacin, riboflavin, biotin, and folic acid (Antony Ceasar et al., 2018; Obilana and Manyasa, 2002). A comparative nutritional analysis with other cereals and millets is provided in **Supplementary Table 1**. Among all cereals, finger millet possesses the highest calcium content, approximately 344 mg per 100 g, which is a critical factor in bone mineralization, skeletal health, and the prevention of osteoporosis (Maharajan et al., 2021; Puranik et al., 2017). Additionally, it contains approximately 8.9 mg/100 g of iron, which supports hemoglobin synthesis and reduces the risk of anemia, and 146 mg/100 g of magnesium, essential for cardiovascular health, blood pressure regulation, and enzymatic function (Kumar and Khare, 2016). Notably, finger millet is an exceptionally abundant reservoir of antioxidants, specifically phenolic compounds. These compounds mitigate oxidative stress and have the potential to defend against chronic diseases such as cardiovascular disorders and type 2 diabetes (Devi et al., 2014; Saleh et al., 2013). The diverse health benefits associated with finger millet consumption are summarized in **Figure 1**. Its nutritional profile positions it as a promising functional grain for modern diets. Understanding these benefits of finger millet is the first step toward incorporating traditional knowledge into modern health practices.

### 2.1 Genetic resources of finger millet

Crop genetic resources (CGRs) form the foundation for crop improvement and adaptation to changing environmental conditions. They provide the raw genetic material necessary for the breeding of superior crop varieties with higher yield potential, improved resistance to pests and diseases, enhanced tolerance to abiotic stresses, and the capacity to support long-term agricultural sustainability. Currently, global gene banks collectively conserve over 38,000 accessions of finger millet, with significant holdings maintained by countries such as India, Ethiopia, Kenya, Zambia and Uganda (Vetriventhan et al., 2020). India boasts the most extensive collections, including 10,507 accessions conserved at the ICAR-National Bureau of Plant Genetic Resources (ICAR-NBPGR) in New Delhi under long-term storage, and 6257 accessions maintained at the All India Coordinated Small MilletsImprovement Project (AICSMIP) in Bangalore under medium-term conditions (Vetriventhan et al., 2020).

The International Crops Research Institute for the Semi-Arid Tropics (ICRISAT), located in Hyderabad, India, manages an additional 5957 finger millet accessions, representing a global collection of eight species: *E. coracana*, *E. africana*, *E. indica*, *E. multiflora*, *E. tristachya*, *E. floccifolia*, *E. kigeziensis*, and *E. jaegeri* (Sood et al., 2019). Further notable collections include 2,875 accessions at the Kenya Agricultural and Livestock Research Organization (KALRO) in Muguga, 2,156 accessions at the Ethiopian Biodiversity Institute (EBI) in Addis Ababa, and 1452 accessions maintained by the USDA Agricultural Research Station in Griffin, Georgia, USA. A summary of these global germplasm repositories are presented in **Table 1**. This comprehensive germplasm resource provides a valuable foundation for identifying and utilizing beneficial alleles associated with agronomic traits, stress resilience, and nutritional quality. Furthermore, it highlights opportunities to enrich existing collections by incorporating weedy and wild relatives that harbor adaptive genes conferring resistance to biotic and abiotic factors, thereby strengthening future breeding efforts.

**Table 1:** Overview of finger millet germplasms and major gene banks (Soodet al., 2019; Gebreyohannes et al., 2024) ICRISAT Genebank (http://genebank.icrisat.org/; accessed on April 2, 2026).

Name of institute/organization	Country	Total accessions	Type of collections
ICAR-National Bureau of Plant Genetic Resources, New Delhi	India	10,507	Indigenous collections, breeding lines, a few exotics and wild relatives
International Crops Research Institute for the Semi- Arid Tropics (ICRISAT), Patancheru	India	7519	Exotic material, global collection including landraces, new cultivars, breeding lines and wild species
All India Coordinated Small Millets Improvement Project, Bengaluru	India	6257	Indigenous collections, breeding lines, few exotic and wild relatives
Kenya Agricultural and Livestock Research Organization (KALRO), Muguga	Kenya	2875	Both indigenous and exotic collections, including wild relatives and breeding lines
Ethiopian Biodiversity Institute (EBI), Addis Ababa	Ethiopia	2156	Indigenous collections, including wild relatives
Agricultural Research Station of the USDA in Griffin, Georgia	USA	1452	Accessions from 11 countries and wild species
Serene Agricultural and Animal Production Research Institute, Soroti	Uganda	1231	Indigenous collections
SADC Plant genetic Resources Centre, Lusaka	Zambia	1037	Indigenous collections
Nepal Agricultural Research Council, Kathmandu	Nepal	869	Indigenous collections
National Centre for Genetic Resources Preservation, Fort Collins	USA	702	–
National Institute of Agrobiological Sciences, Kannondai	Japan	565	–
Mt. Makulu Central Research Station, Chilanga	Zambia	390	–
Institute of Crop Germplasm Resources, Chinese Academy of Agricultural Sciences, Beijing	China	300	Both indigenous and exotic collections

## 3 Physiological and biochemical responses of finger millet to abiotic stresses

### 3.1 Drought stress tolerance

Drought stress exerts a profound negative impact on the finger millet yield, with losses reportedly approaching 61% under severe conditions (Mirza and Marla, 2019). The magnitude of yield reduction in finger millet strongly depends on the developmental stage at which drought occurs, as well as the severity and duration of the stress episode. Exposure to drought elicits a suite of physiological and biochemical alterations in finger millet plants. Growth of both roots and shoots is markedly suppressed, as evidenced by reductions in fresh and dry biomass and overall length (Mundada et al., 2020). During the early seedling phase, drought significantly impairs seed germination, establishment, and coleoptile elongation (Bangari et al., 2023; Rakkammal et al., 2023). Adaptive responses frequently observed in finger millet under drought conditions include pronounced leaf rolling, reduced plant height, decreased leaf area, collectively reflecting the plant’s efforts to conserve water and enhance survival (Bhatt et al., 2011).

The assessment of leaf relative water content (RWC) serves as an indispensable indicator of plant water status, particularly under drought conditions. Drought-tolerant finger millet genotypes deploy a range of adaptive strategies to withstand or evade the deleterious effects of water deficit. Notably, increased RWC in these varieties constitutes a crucial mechanism for conserving cellular hydration in drought stress (Panda et al., 2023; Mundada et al., 2020). However, the maintenance of elevated RWC often necessitates prolonged stomatal closure, which, while limiting transpirational water loss, results in diminished photosynthetic rates and only temporarily sustains plant function. Drought conditions further compromise photosynthetic capacity by impairing the operation of both photosystems I and II (PSI and PSII), disrupting Rubisco activity, and inhibiting ATP synthesis. Consequently, the contents of photosynthetic pigments, including chlorophylls and carotenoids, are significantly reduced under prolonged water deficit (Panda et al., 2023; Mundada et al., 2020; Mukami et al., 2019). Beyond these morpho-physiological adjustments, finger millet plants elicit an array of biochemical adaptations to mitigate drought-induced injury. Central to this response is the enhanced synthesis and accumulation of osmoprotectants (glycine betaine, proline) and various sugars (Raza et al., 2024). These metabolites play pivotal roles in sustaining cellular osmotic equilibrium, safeguarding protein and membrane integrity, and stabilizing subcellular structures during episodes of drought (Panda et al., 2023; Mundada et al., 2020). Their accumulation thus underpins a multifaceted defense system, enhancing the plant’s resilience and safeguarding its productivity under adverse environmental conditions.

Furthermore, drought stress invariably induces the accumulation of ROS, including superoxide (O_2_^-^) and hydrogen peroxide (H_2_O_2_), which can cause severe oxidative damage to cellular components (Mundada et al., 2020; Satish et al., 2018; Bhatt et al., 2011). The resultant elevation in ROS disrupts membrane integrity and induces peroxidative injury to proteins and lipids, giving rise to increased malondialdehyde (MDA) levels and heightened electrolyte leakage (EL) (Panda et al., 2023; Mundada et al., 2020). Notably, investigations assessing the impact of drought stress on finger millet cultivars have revealed that tolerant genotypes manifest substantially enhanced activities of antioxidant enzymes, including catalase (CAT), superoxide dismutase (SOD), glutathione peroxidase (GPX), glutathione reductase (GR), and ascorbate peroxidase (APX), in both root and shoot tissues (Panda et al., 2023; Mundada et al., 2020; Bhatt et al., 2011). In stark contrast, this antioxidative defense response is markedly diminished in susceptible finger millet cultivars, which instead accumulate higher levels of H_2_O_2_, MDA, and exhibit pronounced electrolyte leakage in both roots and shoots. Collectively, these findings underscore the pivotal role of robust antioxidative mechanisms in conferring drought tolerance and safeguarding cellular homeostasis under water-deficit stress (Panda et al., 2023; Mundada et al., 2020; Bhatt et al., 2011).

### 3.2 Salt stress tolerance

Salinity stress remains a critical constraint on global agricultural productivity, adversely impacting approximately 20% of cultivated land and substantially reducing crop yields (Muthuramalingam et al., 2022; Rathinapriya et al., 2020). This abiotic stress arises from the excessive accumulation of soluble salts, such as sodium chloride (NaCl), magnesium chloride (MgCl_2_), and calcium chloride (CaCl_2_) in the soil, surpassing the threshold of plant tolerance (Sarma et al., 2023). Exposure to elevated salinity disrupts plant water uptake due to osmotic stress, wherein reduced water potential at the root-soil interface limits water absorption and perturbs essential physiological and biochemical processes (Munns, 2005; Gupta and Huang, 2014). In parallel, ionic stress emerges from the excessive intracellular accumulation of sodium ions (Na^+^), particularly in older tissues, where salt accumulation exerts cytotoxic effects. Elevated Na^+^ concentrations in the cytoplasm and vacuole impair enzyme function, disturb nutrient homeostasis, and initiate programmed cell death (Munns and Tester, 2008). This dual action of osmotic and ionic stress compromises metabolic integrity and cellular viability. In finger millet, salinity stress leads to pronounced reductions in growth, productivity, and grain quality, posing a significant limitation to its agronomic potential (Mahadik and Kumudini, 2020). The severity of these effects is strongly influenced by the salt concentration, exposure period, and the stage of plant development at which stress occurs. Early-stage exposure tends to inhibit germination and seedling establishment, while stress during reproductive phases drastically impairs grain filling and yield components (Yadav et al., 2019).

Salt stress profoundly impairs the germination, establishment, and terminal development of finger millet seeds (Mbinda and Mukami, 2021). The detrimental effects extend to essential physiological and biochemical processes, disturbing the delicate balance of sodium and potassium ions, and altering proline accumulation, chlorophyll concentration, water content, and the levels of MDA and H_2_O_2_. Salt stress also changes the balance between total protein content and reducing sugars in plant tissues (Mukami et al., 2020; Dugasa et al., 2019; Sarabi et al., 2017; Kumar and Khare, 2016; Hema et al., 2014). Exposure to salinity during the early stages of seedling development causes a marked increase in mortality among salt-sensitive finger millet genotypes and simultaneously suppresses germination rates (Mukami et al., 2020). Beyond these early impacts, salt stress disrupts both the architecture and functionality of chloroplasts, diminishing photosynthetic efficiency, a decline that constrains carbohydrate synthesis and inexorably limits plant growth and final yield (Mukami et al., 2020; Hameed et al., 2021). A concurrent depletion of chlorophyll and carotenoids further underscores the severity of salt-induced injury in finger millet leaves (Mukami et al., 2020; Satish et al., 2016). Notably, salt-tolerant finger millet cultivars often sustain or even elevate chlorophyll levels under saline regimes, in stark contrast to the pronounced reductions observed in sensitive counterparts (Mukami et al., 2020; Mahadik and Kumudini, 2020).

Like many other plant species, finger millet has evolved into a repertoire of sophisticated defense mechanisms to mitigate the harmful consequences of salt exposure. These protective strategies include the enhanced synthesis of antioxidants to scavenge ROS, the meticulous regulation of ion homeostasis and compartmentalization, as well as the modulation of osmolyte accumulation to sustain osmotic balance. Salinity stress induces the accumulation of crucial osmoprotectants such as glycine betaine, proline, and various soluble sugars (Mukami et al., 2020; Satish et al., 2016). These compounds play vital roles in preserving cellular water potential and alleviating osmotic imbalance. Notably, research has demonstrated that finger millet possesses a remarkable capacity for proline accumulation under salt stress conditions, which contributes significantly to stabilizing cellular structures and maintaining turgor pressure (Mukami et al., 2020; Satish et al., 2016). Moreover, salt-tolerant finger millet genotypes exhibit higher levels of proline and carbohydrates, reflecting improved osmotic adjustment and metabolic adaptation under salinity stress (Mukami et al., 2020; Vijayalakshmi et al., 2014).

Plants maintain ion homeostasis under salt stress by finely regulating the transport of Na^+^ and Cl^-^ ions in the roots, thereby preventing excessive accumulation of these ions in the foliage and enhancing salinity tolerance. This process involves the selective removal of Na^+^ from the xylem and its transport back into the rhizosphere. Inefficient Na^+^ exclusion can lead to premature plant death (Rajendran et al., 2009). In finger millet, the extent of salt tolerance is closely linked with the concentration of Na^+^ in the leaves; salt-sensitive genotypes typically amass higher levels, resulting in greater susceptibility (Mukami et al., 2020). For optimal cellular function, plants must sustain a low cytosolic Na^+^/K^+^ ratio, as perturbations in this balance disrupt essential physiological activities (Rahman et al., 2014). When exposed to salinity, salt-tolerant finger millet varieties exhibit markedly reduced Na^+^ accumulation in aerial tissues compared to their sensitive counterparts (Rahman et al., 2014; Vijayalakshmi et al., 2014; Mukami et al., 2020). Notably, the salt-tolerant cultivar Trichy 1 demonstrates a remarkable capacity to regulate Na^+^ uptake, transiently allowing Na^+^ entry into the cytosol before efficiently sequestered into vacuoles, thus minimizing cytoplasmic toxicity. In contrast, this exclusion mechanism is compromised in the salt-sensitive CO-12 variety (Rahman et al., 2014). Moreover, salt-tolerant finger millet genotypes consistently preserve elevated K^+^ levels, which is critical for maintaining cellular integrity and supporting normal physiological processes (Rahman et al., 2014; Vijayalakshmi et al., 2014; Mukami et al., 2020). The enhanced activities of key antioxidant enzymes (CAT, SOD, APX, GPX, and GR) in the leaf tissues effectively mitigate oxidative damage in finger millet under salinity stress (Anjaneyulu et al., 2014).

### 3.3 Temperature stress tolerance in finger millet

Finger millet demonstrates notable resilience to both heat and cold stresses through a spectrum of physiological, biochemical, and molecular mechanisms. While finger millet varieties typically thrive at temperatures up to 36 °C, several summer-adapted accessions exhibit tolerance even at extreme temperatures ranging from 42 °C to 44 °C. Nevertheless, ambient temperatures exceeding 32 °C begins to impair growth and flowering, and sustained exposure above 35 °C induces pronounced heat stress, manifesting in reduced gametogenesis, anthesis failure, lowered seed number per panicle, and a significant decline in overall seed yield (Opole et al., 2018). To counteract heat stress (HS), finger millet orchestrates a multifaceted defense response. One pivotal strategy involves the synthesis of heat shock proteins (HSPs), the molecular chaperones that facilitate proper protein folding and prevent aggregation of heat-denatured proteins. These HSPs, alongside other heat-responsive transcription factors (TFs) and genes, serve as central regulators in the cellular adaptation to thermal stress, enhancing thermotolerance at the transcriptional and post-transcriptional levels (Goyal et al., 2024). Furthermore, finger millet bolsters its antioxidant defense machinery, particularly through the activity of CAT, to detoxify excess heat-induced ROS and prevent oxidative damage to membranes and organelles (Hasanuzzaman et al., 2020; Numan et al., 2021). In addition to molecular responses, finger millet adopts several morpho-physiological strategies to withstand elevated temperatures. These include alterations in carbohydrate metabolism, enabling improved energy supply under stress, and leaf morphological modifications, such as changes in leaf angle and orientation, to reduce solar radiation absorption and minimize transpiration to save water (Maharajan et al., 2021). Conversely, under cold stress, finger millet initiates adaptive responses to maintain cellular integrity and metabolic stability. A key strategy involves modifying the lipid composition of cell membranes to preserve membrane fluidity and prevent ice-crystal-induced mechanical damage. Furthermore, chloroplast rearrangement under low temperatures is observed as a photoprotective mechanism to optimize light capture and preserve photosynthetic efficiency (Maai et al., 2020). Collectively, these findings highlight finger millet’s capacity to adapt to temperature extremes via both short-term physiological plasticity and long-term genetic modulation, positioning it as a promising climate-resilient crop in the face of global temperature fluctuations.

### 3.4 Heavy metal stress tolerance

Heavy metal (HM) contamination in agricultural soils severely compromises crop yield and quality while posing substantial risks to environmental sustainability and human health (Rahman et al., 2019). In plants, HM exposure induces membrane destabilization and uncontrolled production of ROS, such as hydroxyl radicals (^•^OH), H_2_O_2_, and superoxide anions (O_2_•−) (Pandian et al., 2020a). These ROS act as potent oxidants, triggering widespread oxidative damage, including oxidation of lipids and proteins, DNA, and RNA degradation, and enzymatic inhibition, culminating in cellular dysfunction and programmed cell death (Sharma et al., 2019). Among various heavy metals, nickel (Ni) is particularly phytotoxic at elevated concentrations, disrupting key physiological processes and impairing plant metabolism (Kazemi et al., 2010). Finger millet employs a multifaceted defense strategy to counteract HM toxicity, encompassing osmolyte accumulation, antioxidant enzyme activation, and transcriptional reprogramming. One of the primary adaptive responses involves proline accumulation in both root and shoot tissues. This osmoprotectant stabilizes proteins and membranes, mitigates oxidative injury, and acts as a ROS scavenger, thereby enhancing cellular resilience under HM-induced stress (Rakkammal et al., 2024a). Transcriptome studies revealed that finger millet undergoes dynamic differential gene expression (DGE) in response to heavy metal exposure. For instance, RNA-Seq profiling under aluminum (Al) stress revealed upregulation of key candidate genes implicated in detoxification and tolerance, including *carboxypeptidase (SOL1)*, *Heavy Metal ATPase 3* (HMA3), and several transcription factor families such as *APETALA2 (AP2), basic Leucine Zipper (bZIP), Cys3His zinc finger (C3H)*, and *WRKY* (Brhane et al., 2022). Several of these genes are tightly linked to proline biosynthesis and catabolism, and their expression patterns are modulated by Al concentration. This transcriptional plasticity underscores finger millet’s capacity to orchestrate a coordinated molecular defense under HM stress. Additionally, the antioxidant defense system plays a pivotal role in ROS detoxification during HM exposure. Finger millet stimulates the activity of enzymatic antioxidants such as SOD, CAT, and GR, which collectively maintain ROS homeostasis and mitigate oxidative damage. This enzymatic reinforcement is central to finger millet’s physiological tolerance against HM-induced oxidative stress (Rakkammal et al., 2024a). Emerging evidence also supports the use of exogenous plant growth regulators as an auxiliary strategy to bolster HM stress tolerance (Khan et al., 2024). These compounds may amplify endogenous defense pathways, enhance antioxidant enzyme expression, and improve metal sequestration mechanisms. Altogether, these integrated responses, ranging from osmolyte and antioxidant accumulation to transcriptional reprogramming, highlight finger millet’s robust natural defense arsenal and its potential as a resilient crop model for cultivation in HM-contaminated soils.

### 3.5 Antioxidant-mediated abiotic stress tolerance in finger millet

Stress factors such as drought, salinity, heat stress, and heavy metal toxicity induce the overproduction and accumulation of ROS in plant cells, resulting in oxidative stress (Rahman et al., 2024). When ROS generation exceeds the scavenging capacity of the antioxidant system, excess ROS rapidly accumulates and diffuse across cellular compartments, inflicting widespread oxidative damage. Elevated ROS disrupts physiological, biochemical, and molecular processes, ultimately leading to cellular dysfunction and programmed cell death (Sharma et al., 2022). The primary sites of ROS production in plant cells are metabolically active organelles such as mitochondria, peroxisomes, and chloroplasts, where energy metabolism inherently generates ROS as byproducts. Key ROS molecules include the hydroxyl radical (^•^OH), superoxide anion (O_2_^-^), hydrogen peroxide (H_2_O_2_), and singlet oxygen (^1^O_2_), each varying in half-life and redox potential (Miller et al., 2010). These species initiate lipid peroxidation, DNA strand breaks, and protein oxidation, disrupting membranes and metabolic enzymes, and triggering programmed cell death (PCD) (Miller et al., 2010).

In finger millet, exposure to multiple abiotic stresses intensifies ROS accumulation, contributing to reduced seed germination, stunted growth, and compromised yield. Notably, the magnitude of ROS-induced damage varies across developmental stages, tissue types, and genotypes. Under drought stress, the susceptible cultivar PES400 exhibited sharp increases in leaf MDA (49.87%), H_2_O_2_ (147%), and electrolyte leakage (EL) (480%), hallmarks of oxidative injury (Bhatt et al., 2011). Severe osmotic stress induced by 600 mM mannitol and 25% Polyethylene glycol (PEG) further elevated MDA content to 7.23 µmol g^-1^ FW in leaf tissues of sensitive genotypes (Mundada et al., 2020; Mukami et al., 2019). Likewise, 20% PEG treatment increased MDA levels by over 65% in root and shoot tissues of the susceptible IC49940 genotype (Rakkammal et al., 2023). Furthermore, salinity stress also triggers severe oxidative membrane damage. For instance, four sensitive finger millet cultivars (GBK043050, GBK043122, GBK043124, and GBK043128) exhibited significantly higher MDA levels under 300 mM NaCl treatment (Mukami et al., 2020). Consistent with these findings, other studies documented increased H_2_O_2_ and MDA content in leaf and root tissues under salt stress (Mahadik and Kumudini, 2020; Rakkammal et al., 2024a). Heat stress was similarly associated with increased H_2_O_2_ and O_2_^-^ radicals in finger millet seedlings (Solanke et al., 2018). Furthermore, exposure to heavy metals, including arsenic, nickel, and their combination, also led to pronounced elevation of H_2_O_2_, O_2_^-^, and MDA levels, reflecting intensified oxidative stress (Kotapati et al., 2017; Rakkammal et al., 2024a).

Plants have evolved sophisticated antioxidant defense systems to neutralize ROS and mitigate oxidative damage under stress conditions. The tight regulation of ROS homeostasis is maintained through the coordinated activity of enzymatic and non-enzymatic antioxidant components (Caverzan et al., 2016). The enzymatic antioxidant machinery includes key enzymes such as SOD, CAT, APX, GR, GPX, monodehydroascorbate reductase (MDHAR), and dehydroascorbate reductase (DHAR). In parallel, non-enzymatic antioxidants, including ascorbate (AsA), glutathione (GSH), carotenoids, tocopherols, and various phenolic compounds, complement this system by scavenging ROS directly or serving as cofactors in redox cycling. SOD (EC 1.15.1.1), a metalloenzyme, is pivotal in the dismutation of superoxide anions (O_2_^-^) into hydrogen peroxide (H_2_O_2_) and molecular oxygen (O_2_). This detoxification step prevents the formation of highly reactive hydroxyl radicals (^•^OH) via the Haber–Weiss reaction (Gill and Tuteja, 2010).

Plant SODs are classified into three isoenzymes based on their metal cofactors: iron-SOD (Fe-SOD), copper/zinc-SOD (CuZn-SOD), and manganese-SOD (Mn-SOD). These isoforms are localized to distinct cellular compartments, including chloroplasts, mitochondria, peroxisomes, the nucleus, and the apoplast (Mittler, 2002). CAT (EC 1.11.1.6), a tetrameric iron-containing enzyme, catalyzes the decomposition of H_2_O_2_ into water and oxygen, thereby protecting cells from hydrogen peroxide toxicity. CAT enzymes are categorized into three functional groups: Group I (monofunctional catalase), Group II (catalase-peroxidase), and Group III (non-heme catalase) (Loewen et al., 2004). Most higher plants possess Group I catalases, which are widely distributed in the cytosol, chloroplasts, and mitochondria (Kesawat et al., 2023). APX (EC 1.11.1.11) functions as a key H_2_O_2_-scavenging enzyme in the Asada-Halliwell-Foyer pathway, where it utilizes AsA as an electron donor to convert H_2_O_2_ into water and DHA (Foyer and Noctor, 2011). In the absence of AsA, APX activity is significantly reduced, underscoring its dependence on the ascorbate pool for optimal function. APX isoforms are distributed across multiple organelles, including the cytosol (cAPX), chloroplasts (chlAPX), mitochondria (mtAPX), and peroxisomes/glyoxysomes (mAPX) (Lazzarotto et al., 2011).

GR (EC 1.6.4.2) is a key antioxidant enzyme belonging to the flavoprotein oxidoreductase class, catalyzing the reduction of GSSG to GSH using NADPH as an electron donor. This reaction is vital for maintaining cellular redox balance and sustaining the pool of reduced GSH, which is essential for ROS detoxification. GR isoforms are abundantly localized in the chloroplasts, cytosol, and mitochondria, reflecting their critical function in diverse subcellular compartments (Creissen and Mullineaux, 2002). GPX (EC 1.11.1.7) is another pivotal enzyme that contains a cysteine residue in its active site and catalyzes the reduction of H_2_O_2_ and lipid hydroperoxides to water and corresponding alcohols, respectively (Madhu et al., 2023). GPX utilizes GSH as a co-substrate, resulting in its oxidation to GSSG during the detoxification process. While GPX is predominantly chloroplastic, its isoforms are also present in mitochondria, cytosol, and peroxisomes, underscoring its ubiquitous role in cellular redox regulation (Xiao et al., 2008). The efficacy and responsiveness of these enzymatic antioxidants vary significantly depending on genotype, developmental stage, tissue specificity, and the time span and degree of exposure to abiotic stress. In 1, antioxidant enzymes, including CAT, SOD, GR, APX, and GPX, form the core machinery for ROS detoxification under stress exposure (**Table 2**). Under drought conditions, leaf tissues of drought-tolerant finger millet genotype PR202 showed significantly increased activities of SOD (47%), APX (56%), GR (45%), and CAT (13%), along with reduced levels of MDA, H_2_O_2_, and electrolyte leakage compared to the susceptible genotype PES400 (Bhatt et al., 2011). Likewise, in the drought-tolerant genotype IC 87469, PEG-induced osmotic stress triggered >51% increases in SOD activity in both leaf and root tissues, CAT activity (>40% in leaf, >50% in root), GR activity (>50%), and GPX activity (>50%), while minimizing photosynthetic pigment loss and membrane damage (Rakkammal et al., 2023). Mundada et al., 2020 further demonstrated that PEG-induced drought stress increased SOD activity (2.0-fold), CAT (1.68-fold), and APX (3.09-fold) in the shoots of a drought-tolerant finger millet cultivar. In the presence of heavy metal stress (As, Ni, and As+Ni), finger millet roots exhibited markedly elevated antioxidant enzyme activity-SOD (94.9%, 89.4%, 94.4%), CAT (88%, 82.4%, 90.3%), and GR (>90%)-with a preferential accumulation of metals in roots over shoots, suggesting a localized antioxidant response (Rakkammal et al., 2024a).

**Table 2:** Role of antioxidant enzymes and other metabolites in abiotic stress tolerance in finger millet.

Abiotic stress	FM variety/accession	Treatment and duration	Key findings	References
Drought	PR202, VL146, VL315, PES400 and VR708	45 days old plants were subjected to severe drought (45% FC) for 6 days	Tolerant genotype (PR202) showed increased SOD, APX, GR, and CAT activity; lower MDA, H2O2 and EL	Bhatt et al. (2011)
Drought	-	FM seedlings were grown under PEG 5%, 10%, 15%, 20% and 25% for 14 days	Increased SOD, CAT, APX, and GPX activities	Mundada et al. (2020)
Drought	IC 87469 and IC 49940	21 days old plants were exposed to 5%, 10%, 15% and 20% PEG for 7 days	Tolerant genotype (IC 87469): increased SOD, CAT, GR, GPX; decreased MDA	Rakkammal et al. (2023)
Salt	CO(Ra)-14	50, 100, 150 and 200 mM salt for 15 days	Increased cell-bound phenolic compounds	Satish et al., 2016
Salt	ST-JA-SM; ST-JA-WA	200 mM NaCl	Enhanced osmolyte and carotenoid accumulation; increased SOD, CAT, APX, and GPX activity	Shaikh et al., 2025
Salt	IE 518 and IE 405	0, 15, and 30 dSm−1 for 7 days	Enhanced accumulation of mannose, melibiose, L-Proline and GABA	Mondal and Jespersen, 2025
Low temperature	Yukijirushi	5, 10 and 25 °C	Declined Fv/Fm; increased ABA accumulation in response to light; induced chloroplast arrangement	Maai, et al., 2020
Arsenic and Nickel	CO-Ra-14	14 days old plants were exposed to As, Ni and As + Ni for 7 days	Increased activities of SOD, CAT, and GR, while decreased MDA and H2O2 content and EL in root tissues	Rakkammal et al. (2024a)
Nickel	Sri Chaitanya VR-847	0.5 mol L−1 Ni for 7 days	CAT, SOD, APX, SA functionally interact to mediate nickel toxicity resilience	Kotapati et al., 2017
Nickel	PR 202, PES 110, GPU 28, GPU 20, and GPU 66	0, 10, 15, 20, 25, 30, and 40 ppm for 15 days	Enhanced POD and SOD activity, while declined CAT activity	Gupta et al., 2017
Aluminum	Accessions 215836,215845, 229722, 212462, 215804, and 238323	100 μM Al for 10 days	Enhanced proline, auxin biosynthetic gene expression; cytoplasmic Al removed and sequestered in vacuole	Brhane et al., 2022

**Figure 2:**
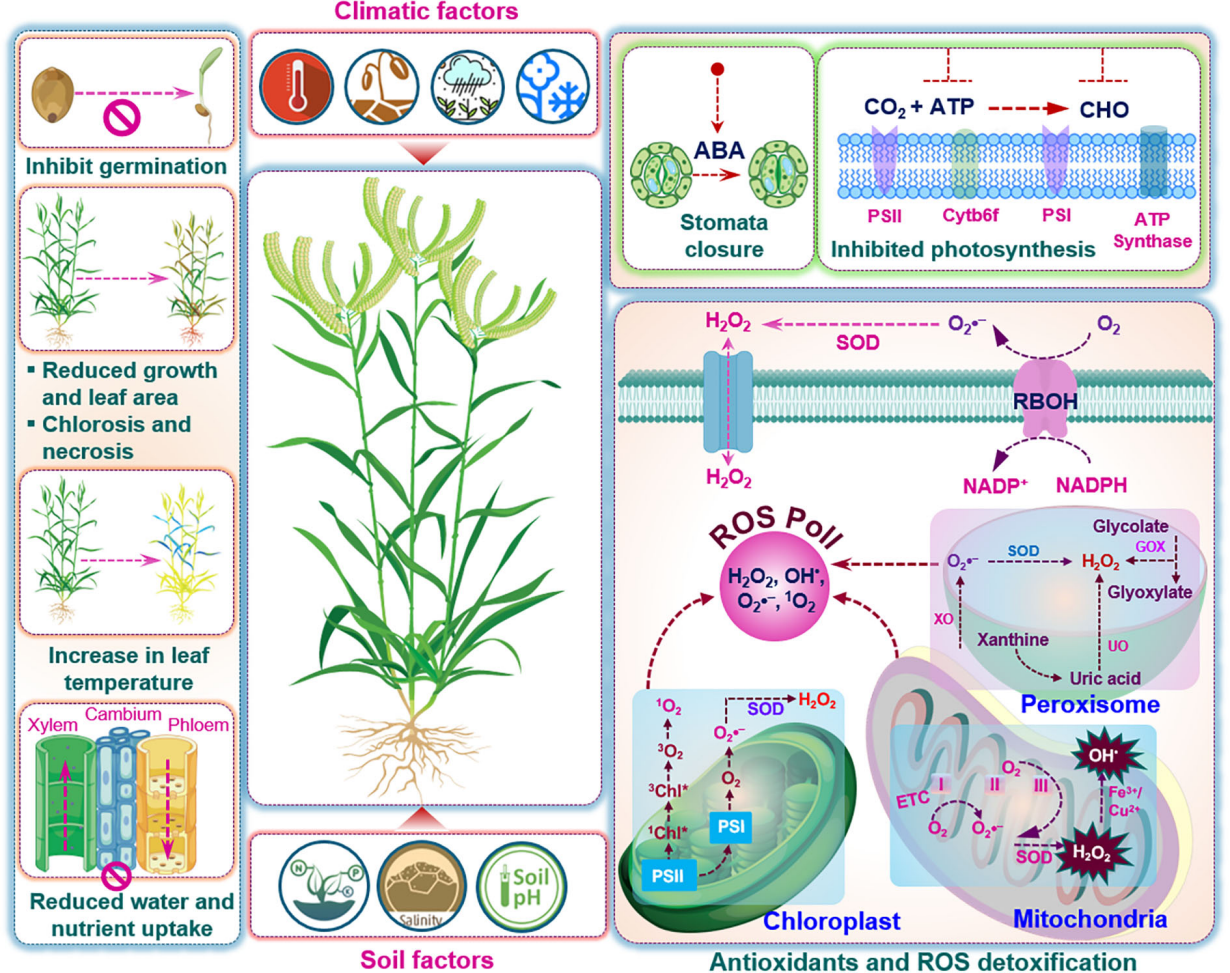
Environmental constraints and oxidative stress responses in finger millet. The diagram summarizes how climatic and edaphic factors impair growth and activate ROS, reactive oxygen species metabolism in finger millet. Climatic drivers (temperature extremes, drought, flooding, and cold) together with soil factors (salinity, nutrient imbalance, and suboptimal pH) inhibit germination, reduce vegetative growth and leaf expansion, induce chlorosis and necrosis, increase leaf temperature, and restrict water and nutrient uptake through their effects on vascular tissues. Stress perception in leaves promotes ABA, abscisic acid accumulation, stomatal closure, and inhibition of photosynthetic electron transport and ATP synthesis. Plasma-membrane NADPH oxidases and organellar metabolism in chloroplasts, mitochondria, and peroxisomes together contribute to an increased cellular ROS pool, including superoxide, hydrogen peroxide, hydroxyl radical, and singlet oxygen. Antioxidant enzymes such as superoxide dismutase and associated detoxification pathways in these organelles act to restore redox balance and limit ROS-induced damage.ROS, reactive oxygen species; ABA, abscisic acid; CO_2,_ carbon dioxide, ATP. Adenosine triphosphate, CHO, aldehyde functional group; PS, photosystem; Cytb6f: cytochrome b6f protein complex; H_2_O_2_, hydrogen peroxide, SOD, superoxide dismutase; GOX, glyoxalate; O_2_, oxygen; O_2_^•−^, superoxide radical anion; Chl, chlorophyll; Fe^3+^, ferric ion, an iron atom; Cu^2+^, copper ion.

The enzymatic ROS- detoxification system plays an indispensable role in safeguarding finger millet against oxidative damage caused by abiotic stresses. **Figure 2** illustrates the physiological adaptations and antioxidant strategies deployed by finger millet under stress conditions. While low levels of ROS are increasingly recognized as secondary messengers that modulate stress-responsive gene expression, extensive research exploring this specific signaling role in finger millet remains limited. Integrative omics approaches are urgently needed to elucidate the oxidative stress response and unlock genetic potential for breeding finger millet genotypes with enhanced multi-stress resilience. A comparative summary of key studies on abiotic stress tolerance in finger millet is presented in **Table 3**.

**Table 3:** Summary of key studies on abiotic stress tolerance in finger millet.

Study	Stress type	Approach	Key findings
Bhatt et al., 2011	Drought	Physiological and biochemical analysis	Enhanced antioxidant enzyme activity in tolerant genotypes
Mundada et al., 2020	Drought	PEG-induced stress experiments	Increased osmoprotectants and ROS detoxification
Panda et al., 2023	Drought	Physiological assessment	Maintenance of higher relative water content in tolerant genotypes
Rahman et al., 2014	Salinity	Transcriptomic analysis	Identification of genes related to ion transport and stress signaling
Mukami et al., 2020	Salinity	Physiological and biochemical analysis	Altered ion homeostasis and antioxidant responses
Mahadik and Kumudini, 2020	Salinity	PGPR-based study	Improved growth and antioxidant activity under salt stress
Goyal et al., 2024	Heat	RNA-seq transcriptomics	Identification of differentially expressed genes
Li et al., 2021	Drought	Transcriptomics + proteomics	Identification of stress-responsive genes and proteins
Brhane et al., 2022	Heavy metal (Al)	RNA-seq	Upregulation of detoxification-related genes
Rakkammal et al., 2023	Drought	Gene expression profiling	Identification of key drought-responsive genes
Rakkammal et al., 2024a	Heavy metals	Physiological and molecular analysis	Enhanced antioxidant defense and proline accumulation
Chandra et al., 2020	Drought	PGPR application	Improved antioxidant activity and osmolyte accumulation
Mishra et al., 2023	Drought	Nanoparticle application	Improved photosynthesis and stress tolerance

## 4 Molecular strategies of finger millet beyond abiotic stress tolerance

The molecular adaptations of plants to abiotic stress are orchestrated through a highly intricate and multilayered regulatory network. This complexity spans stress perception, signal transduction, transcriptional reprogramming, translational control, and post-translational modification. At the heart of this adaptive machinery lies the concerted interplay of diverse gene families, signaling cascades, and metabolic pathways, all tightly coordinated to ensure survival under adverse environmental conditions. Upon exposure to abiotic stress, plants swiftly activate a suite of stress-responsive genes and regulatory modules that encode proteins critical for physiological adjustment and cellular homeostasis. These include transcription factors, signaling kinases, ion transporters, antioxidant enzymes, and molecular chaperones, each contributing to finely tuned acclimation responses (Ali et al., 2022). The integration of these molecular layers underscores the plant’s remarkable capacity to perceive, decode, and mount targeted defenses against a wide range of abiotic stresses.

### 4.1 Integrated OMICS approach

**Figure 3:**
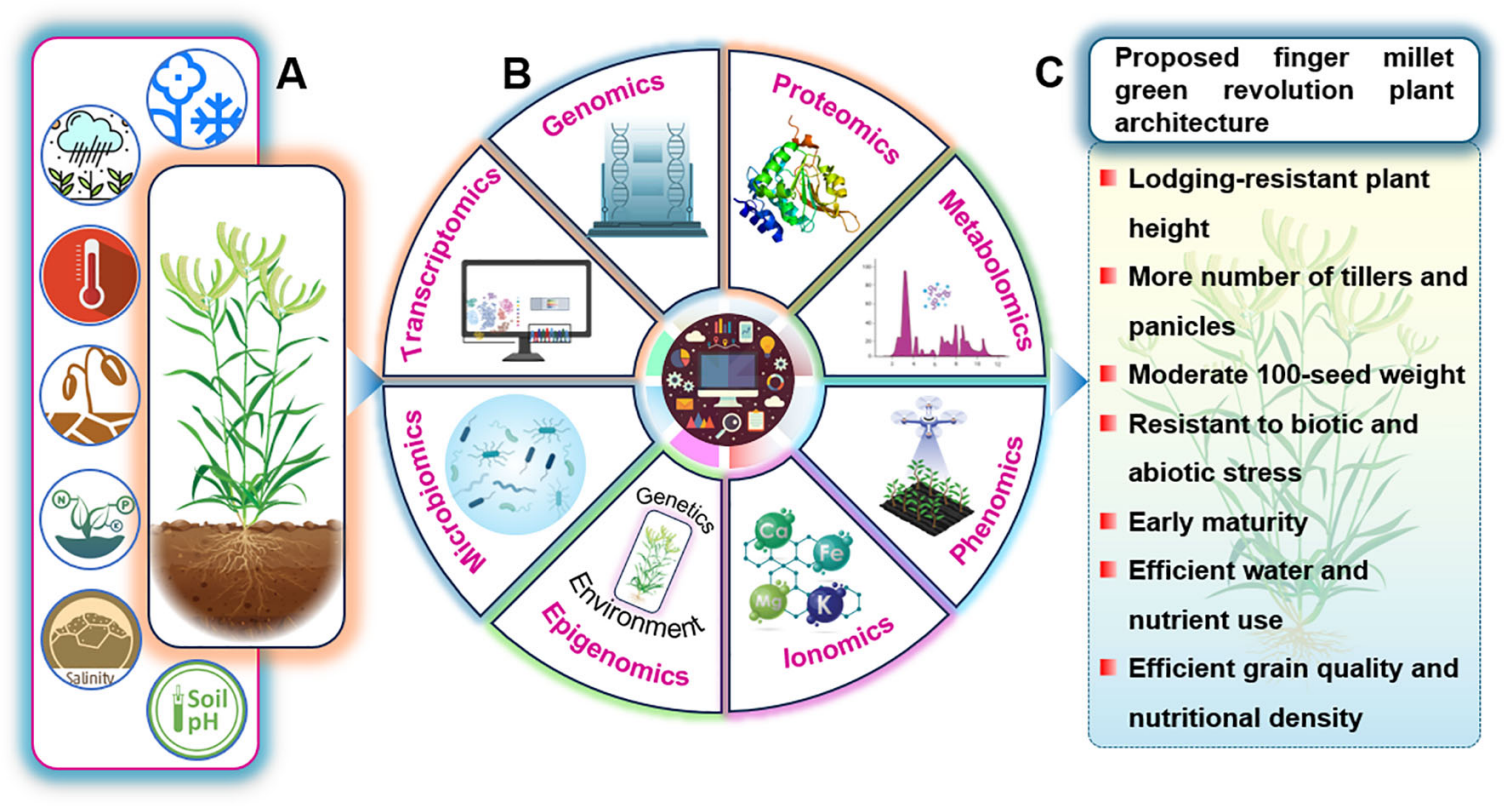
Multi−omics roadmap to a green−revolution–type finger millet ideotype. **(A)** Current finger millet plants grown under suboptimal environments experience multiple climatic and soil constraints, including temperature extremes, drought, waterlogging, salinity and unfavorable soil pH, which together restrict root development, shoot growth and productivity. **(B)** A central multi−omics framework integrating genomics, transcriptomics, proteomics, metabolomics, phenomics, ionomics, epigenomics and microbiomics is used to decode the genetic and environmental determinants of these complex stress responses. **(C)** Insights from these combined data layers are used to define a target “green revolution” plant architecture for finger millet, characterized by lodging−resistant but high−yielding plants with more tillers and panicles, moderate 100−seed weight, broad resistance to biotic and abiotic stresses, early maturity, efficient water and nutrient use and improved grain quality and nutritional density.

Omics technologies have revolutionized the landscape of plant science by providing the methodological precision, technical depth, and interdisciplinary integration is necessary to unravel complex genomic and regulatory networks (Ali et al., 2022). The omics framework encompasses a suite of interconnected disciplines, including genomics (resource generation, functional annotation, genomic selection and gene mapping), transcriptomics (gene expression dynamics and regulatory mechanisms), proteomics (identification of proteins and their functional impact), metabolomics (metabolite profiling and pathway elucidation), phenomics (physiological and phenotypic characterization), and ionomics (elemental profiling and nutrient interactions) (Ali et al., 2022; Muthuramalingam et al., 2022; Rakkammal et al., 2022; Jeyasri et al., 2021; Pandian et al., 2020b). While omics-assisted research has markedly advanced stress-resilience breeding in major crops such as rice, wheat, maize, soybean (*Glycine max*), and tomato (*Solanum lycopersicum*), finger millet remains underexplored, particularly in transcriptomic, metabolomic, and proteomic domains. These next-generation tools are crucial for capturing subtle molecular and physiological changes triggered by genetic variation, nutrient dynamics, and environmental stressors. Integrative multi-omics approaches offer a transformative pathway to decode the stress adaptation landscape of finger millet, facilitating the development of climate-resilient genotypes tailored for future agricultural demands. A conceptual framework illustrating the integration of omics approaches for enhancing abiotic stress tolerance in finger millet is presented in **Figure 3**.

### 4.2 Finger millet genomics

In the early 2000s, a range of molecular markers, such as random amplified polymorphic DNA (RAPD), inter-simple sequence repeats (ISSR), amplified fragment length polymorphisms (AFLP), and expressed sequence tag-derived simple sequence repeats (EST-SSRs) were employed to assess the genetic diversity of finger millet (Gupta et al., 2010; Naga et al., 2012; Babu et al., 2014). These early tools provided foundational insights but were limited in resolution and genome-wide coverage. The rise of next-generation sequencing (NGS) technologies has since transformed the landscape of finger millet genomics, enabling high-resolution exploration of the genome and its response to environmental stresses. Whole-genome sequencing (WGS) efforts on two finger millet genotypes, ML-365 and PR202, significantly expanded genomic resources for the crop (Hittalmani et al., 2017; Hatakeyama et al., 2018). The first draft genome, derived from ML-365 and released in 2017, consisted of 525,759 scaffolds with an average scaffold length of 2,275 bp. Using the *de novo* gene prediction tool Augustus, researchers identified 78,647 non-transposable elements (non-TE) genes and 6,596 TE-associated genes. Functional annotation revealed 2,866 drought-responsive genes, 330 genes related to calcium transport and accumulation, and 146 genes implicated in the C_4_ photosynthetic pathway. Additionally, transcription factor families known to mediate abiotic stress responses, such as ZF-HD, MYC, MYB, WRKY, ABF, AREB, NF-Y, NAC, and GRF, were catalogued in diverse plant species. These genomic datasets are publicly accessible through the National Center for Biotechnology Information (NCBI) under BioSample accessions SAMN04849255 and SAMD00076255 (Hatakeyama et al., 2018; Hittalmani et al., 2017).

A second draft genome for genotype PR202, published by Hatakeyama et al., 2018, comprised 2,387 scaffolds and annotated 62,348 genes, of which 91% were functionally characterized and 96.5% identified as single-copy. This assembly featured a maximum scaffold length of 5 Mb and an N50 of 905.3 kb, indicating a substantial improvement in assembly contiguity and annotation quality over earlier efforts. More recently, a high-quality chromosome-level genome assembly of the Kenyan finger millet cultivar KNE 796 was released by the Devos group at the University of Georgia (Devos et al., 2023). Constructed using the MECAT assembler, which integrates mapping, error correction, and *de novo* assembly, the genome comprises approximately 48,836 high-confidence and 24,176 low-confidence gene models. This comprehensive assembly is now publicly available through the Phytozome database v13 (https://phytozome-next.jgi.doe.gov/info/Ecoracana_v1_1), representing the most advanced genomic resource for finger millet to date (Goodstein et al., 2012). Together, these sequencing efforts have established a robust genomic framework for finger millet. This foundational knowledge base now enables the targeted detection of genes linked to stress adaptation, coupled with the generation of advanced, high-definition molecular markers, and the deployment of genomics-assisted breeding strategies to enhance resilience, productivity, and nutritional quality in this underutilized but climate-resilient crop.

### 4.3 Molecular regulation of stress tolerance by key candidate genes

Several candidate genes associated with drought stress responses have been characterized in finger millet, shedding light on the molecular mechanisms underlying stress adaptation. Gene expression analysis conducted under varying drought intensities measured as 20%, 40%, and 60% of soil field capacity (the maximum water retained by the soil after gravitational drainage) revealed differential expression of key drought-responsive genes, including *farnesyl pyrophosphate synthase* (*FPS*), *basic leucine zipper* (*bZIP*), *transcriptional regulator* (*TR*), *metallothionein* (*MT*), *protein phosphatase 2A* (*PP2A*), *early light-inducible proteins* (*ELIP*), and *farnesylated protein 6* (*FP6*) in finger millet leaf tissues (Parvathi et al., 2013). Among these, *MT*, *bZIP*, *FP6*, *PP2A*, and *TR* exhibited significantly higher expression levels under severe drought stress (60% field capacity), indicating their potential roles in stress mitigation. In a separate study, Bhatt et al. (2013) evaluated the expression of an isoform of ascorbate peroxidase (*Ecapx1*) at the mRNA level under drought stress. The drought-tolerant cultivar PR202 displayed significantly higher *Ecapx1* expression and APX activity compared to the drought-susceptible genotype PES400, suggesting a key role for APX in oxidative stress regulation. More recently, Rakkammal et al. (2023) eight classical drought-responsive genes were identified in the finger millet genome, and their expression profiles were compared between shoot and root tissues of drought-tolerant and drought-susceptible genotypes. These genes included *EcP5CS* (*Δ¹-pyrroline-5-carboxylate synthase*), *EcCAX1* and *EcCAX3* (*cation/proton exchangers*), *EcNAC67* (*no apical meristem domain*), *EcCAT1* (*catalase*), *EcSOD* (*superoxide dismutase*), *EcGR* (*glutathione reductase*), and *EcAPX1 ascorbate peroxidase*). Among these, *EcP5CS*, *EcSOD*, *EcCAT1*, and *EcGR* were highly expressed in drought-tolerant genotypes, implicating their involvement in osmoprotection and ROS detoxification pathways. Collectively, these candidate genes represent promising molecular targets for improving drought tolerance in finger millet through functional genomics and molecular breeding approaches.

### 4.4 Finger millet transcriptomics

Transcriptome profiling provides a comprehensive understanding of gene expressions and associated regulatory frameworks, supporting the detection of key genes involved in abiotic stress tolerance. Depending on species-specific genomic resources and research objectives, a variety of transcriptomic tools have been employed, including Affymetrix GeneChips, expressed sequence tags (ESTs), suppression subtractive hybridization, spotted microarrays, and RNA sequencing (RNA-Seq) (Lowe et al., 2017). Among these, RNA-Seq has become the preferred platform due to its high resolution, cost-effectiveness, and throughput, particularly with the advent of next-generation sequencing technologies (Kamali and Singh, 2022). Although transcriptomic research in finger millet is still limited compared to major cereals, several studies have shed light on the molecular basis of stress tolerance. For instance, Rahman et al. (2014) conducted a transcriptome analysis in two contrasting finger millet genotypes, TRY 1 (salt-tolerant) and CO 12 (salt-sensitive), and identified differentially expressed genes (DEGs) associated with salt stress. These included *Aquaporin (AQP)*, *AP2-domain* (*AP2*), *Catalase isoform A* (*CATA*), *Auxin response factor* (*ARF*), *NAD epimerase* (*NADE*), *Glycosyl transferase 8* (*GT8*), *Na^+^/Ca^2+^ exchanger* (*NCX*), and *Sucrose phosphate synthase* (*SPS*) each of which exhibited elevated expression levels in the tolerant genotype. In another study, transcriptomic profiling of leaf tissues from the drought-tolerant genotype GPU-28 revealed novel regulatory genes such as *serine/threonine protein phosphatase 2A* (*PP2A*), *signal recognition particle receptor α* (*SRPRα*), *farnesyl pyrophosphate synthase* (*FPS*), and *calcineurin B-like interacting protein kinase 31* (*CIPK31*), all implicated in drought response pathways (Parvathi et al., 2019). More recently, Goyal et al. (2024) performed a comparative transcriptome analysis of heat-tolerant (PES-110) and heat-sensitive (KJNS-46) genotypes under heat stress. A total of 684 DEGs were identified, with 494 transcripts upregulated and 190 downregulated in PES-110 compared to KJNS-46. These genes were associated with pathways related to transcriptional regulation, heat shock protein (HSP) expression, water deprivation response, redox homeostasis, and calcium- and kinase-mediated signaling. The emerging transcriptomic datasets in finger millet are increasingly valuable for identifying DEGs and molecular markers responsive to specific abiotic stress conditions. Continued exploitation of these resources will be essential for accelerating the development of stress-resilient finger millet cultivars through marker-assisted selection and functional genomics approaches, ultimately enhancing growth and yield under adverse environmental conditions.

### 4.5 Finger millet proteomics

Proteomics plays a pivotal role in elucidating the functional outputs of gene expression by directly assessing protein abundance, post-translational modifications, and activity (Mustafa and Komatsu, 2021). Unlike transcriptomics, changes in transcript levels do not always correlate with protein expression or function, highlighting the necessity of proteomic approaches for a comprehensive understanding of plant stress responses (Hu et al., 2015). By integrating transcriptomic data, proteomics enables the identification of regulatory mechanisms underlying translation, folding, degradation, and modification of proteins in response to environmental stimuli. Advancements in high-throughput proteomics technologies such as isotope-coded affinity tags (ICATs), isobaric tags for relative and absolute quantitation (iTRAQ), multidimensional protein identification technology (MudPIT), and targeted mass tags (TMTs), have significantly expanded the capacity to profile complex plant proteomes under abiotic stress. While proteomics has advanced considerably in major cereals and other millets, studies in finger millet remain limited. However, a recent integrated transcriptome–proteome analysis by Li et al. (2021) uncovered key genes and proteins associated with drought resilience in finger millet. At the transcriptome level, 80,602 genes exhibited differential expression, while 3,009 differentially expressed proteins (DEPs) were identified. Coordinated expression patterns between DEGs and DEPs highlighted crucial functional networks contributing to drought tolerance. These DEPs were associated with hydrolase activity, oxidoreductase activity, carbohydrate binding, unsaturated fatty acid biosynthesis, and glycosyl bond formation (Li et al., 2021). In another study, the jasmonate ZIM-domain (EcJAZ) protein was functionally investigated in finger millet under methyl jasmonate treatment. The proteomic analysis revealed that *EcJAZ* acts as a central regulator within jasmonate and cross-phytohormone signaling pathways. This protein plays a key role in modulating finger millet responses to environmental stress and developmental cues, thereby contributing to adaptive fitness (Sen et al., 2016).

### 4.6 Metabolomics analysis in finger millet

Metabolic profiling serves as a powerful tool for assessing an organism’s biological and physiological status by examining metabolites, which are the end products of gene expression. In plant systems biology, the integration of metabolomics is crucial for deciphering complex cellular processes and understanding stress-responsive metabolic reprogramming. Owing to the vast chemical diversity and dynamic nature of plant metabolites, the complete size of the plant metabolome remains undefined (Pontarin et al., 2020). To address this complexity, researchers employ advanced analytical platforms such as mass spectrometry (MS)-based approaches and nuclear magnetic resonance (NMR). These include gas chromatography mass spectrometry (GC-MS), capillary electrophoresis mass spectrometry (CE-MS), liquid chromatography mass spectrometry (LC-MS), ultra-high-resolution performance chromatography–mass spectrometry (UHPLC–MS) and flow infusion electrospray ionization high-resolution mass spectrometry (FIE–HRMS) (Kosmacz et al., 2020). Despite growing interest in metabolomics, its application in millets remains limited relative to major cereals, and comprehensive metabolomic profiling of finger millet under abiotic stress conditions are notably lacking. However, preliminary insights are available from related minor millets. For instance, Pan et al. (2021) conducted a comparative metabolite profiling of finger millet varieties (Yugu2 and An04) under salt stress. The tolerant genotype Yugu2 exhibited enhanced regulation of ion channels and antioxidant pathways, with key alterations observed in lysophospholipid, phenylpropanoid, flavonoid, and lignin biosynthesis pathways. Similarly, a GC–MS-based metabolomics study in the tolerant little millet genotype “OLM-203/Tarini” identified 60 metabolites spanning 25 metabolic pathways in response to water deficit stress (Dhawale, 2022). These studies provide valuable reference points for initiating metabolomics research in finger millet under abiotic stress, offering a foundation for identifying stress-responsive metabolic signatures and potential biomarkers for crop improvement.

### 4.7 Finger millet ionomics

Ionomics involves large-scale profiling of the elemental composition within plants, employing high-throughput techniques to detect and quantify mineral nutrients and trace elements across diverse species. This discipline provides critical insights into how elements influence plant biochemistry, physiology, and nutritional status. Analytical methods such as inductively coupled plasma atomic emission spectroscopy (ICP-AES) and inductively coupled plasma mass spectrometry (ICP-MS) are widely used to characterize plant ionomics profiles with high sensitivity and precision (Ali et al., 2022). The elemental composition of plants is shaped by multiple factors, including soil nutrient availability, root architecture, ion uptake capacity, transport dynamics, and the plant’s physiological responses to environmental stimuli (Ali et al., 2021). Several mineral elements, notably calcium, sulfur, magnesium, zinc, iron, selenium, and silicon, are associated with enhancing tolerance against salinity, drought, and heavy metal stress. These elements contribute to stress tolerance through mechanisms such as osmotic adjustment, antioxidative defense activation, membrane stabilization, and detoxification of ROS (Kumari et al., 2022). In finger millet, recent bioinformatics studies have identified and characterized several mineral nutrient transporter proteins, providing foundational knowledge for targeted manipulation of nutrient acquisition and homeostasis under stress conditions (Maharajan et al., 2022). Nevertheless, our current perspective on the synergistic functions of ionomic networks in coordinating stress responses remains limited. Future investigations should aim to elucidate the cross-talk between specific mineral ions and stress signaling pathways, which will be critical for engineering nutrient-efficient and stress-resilient crop genotypes.

### 4.8 Phenomics in finger millet

Phenomics refers to the comprehensive study of all observable plant traits and their interactions with genotype and environment (G×P×E), particularly under defined environmental conditions using high-throughput analytical methods (Yang et al., 2020; Yadav et al., 2022). Phenotypic traits serve as the functional link between a plant’s genetic architecture and its environmental context. However, phenotyping, especially the characterization of complex, quantitative traits associated with stress tolerance, has progressed more slowly than genomic technologies (White et al., 2012). This lag is primarily due to the intricate and dynamic biosynthetic responses that plants deploy under abiotic stress, making high-resolution, large-scale phenotyping a considerable challenge (Yadav et al., 2022). Recent advances in non-invasive phenotyping technologies have facilitated the monitoring of plant growth, morphology, and physiological responses under stress. These include color imaging for biomass assessment, far-infrared imaging for canopy temperature, RGB digital imaging, LIDAR scanning, and magnetic resonance imaging (MRI) for internal structural analysis (Yang et al., 2021). Specific platforms have enabled trait-specific insights; for example, PHENOPSIS is an automated system developed for monitoring drought response in *Arabidopsis thaliana* (Granier et al., 2006). The use of chlorophyll fluorescence imaging has shown success in screening abiotic stress reactions in several crops, including canola (*Brassica napus*), tobacco, and cotton (*Gossypium hirsutum*) (Saranga et al., 2004; Baker, 2008). Infrared Thermography has also been applied to assess stomatal conductance in wheat and barley under salinity stress (Sirault et al., 2009). Similarly, RGB imaging in soil-filled rhizoboxes has enabled shoot and root phenotyping in cereals (Humplík et al., 2015). Despite these technological advancements, there is an urgent need for the integration of deep learning and artificial intelligence algorithms to extract and interpret phenotypic data from the vast and complex datasets generated. Furthermore, the effective coordination and standardization of phenotyping platforms, software pipelines, and data management systems remain key challenges in advancing phenomics for stress resilience research.

## 5 Genes and transcription factors confer abiotic stress tolerance in finger millet

Finger millet is well-known for its exceptional resistance to various abiotic stress conditions, such as drought and salt, as well as for its rich nutritional profile (Sood et al., 2019; Gupta et al., 2017). In recent years, significant efforts have been made to identify and characterize stress-responsive genes and transcription factors in finger millet. A summary of these functionally validated targets including their utility for enhancing abiotic stress tolerance in heterologous plant systems is provided in **Table 4**. The drought stress-responsive gene *EcDehydrin7*, isolated from thedrought-tolerant MR1 genotype of finger millet, was overexpressed in *Nicotiana tabacum* (tobacco), resulting in enhanced drought tolerance in transgenic lines (Singh et al., 2015). Similarly, two transcription factors, such as *EcbZIP60* (basic leucine zipper family) and *EcbHLH57* (basic helix-loop-helix family), were isolated from the GPU-28 finger millet genotype and functionally validated in tobacco. Overexpression of *EcbZIP60* conferred improved drought tolerance, characterized by increased stomatal conductance and photosynthetic efficiency (Babitha et al., 2015a). Moreover, Moreover, *EcbHLH57* overexpression significantly upregulated key stress-inducible genes, including *rd29A*, *rd29B*, *LEA14*, *SOD*, *APX*, *ADH1*, *HSP70*, and *PP2C*, thereby enhancing both drought and salinity tolerance in transgenic tobacco (Babitha et al., 2015b). The *EcNAC67* gene, isolated from the salt-tolerant Trichy-1 genotype of finger millet, was introduced into the high-yielding rice cultivar ASD16. Transgenic rice lines demonstrated enhanced resistance to drought and salinity under controlled greenhouse conditions, with improvements in shoot and root biomass, relative water content, and recovery efficiency, although a slight reduction in grain yield was observed (Rahman et al., 2016). Another key regulator, *EcbZIP17*, encoding an ER membrane-tethered transcription factor, was shown to enhance tolerance to multiple environmental stresses in tobacco. This improvement was linked to the activation of unfolded protein response (UPR)-associated genes including *CRT1*, *BiP*, and *PDIL* (Ramakrishna et al., 2018). Further, overexpression of *EcCaM* (calmodulin) in *Arabidopsis thaliana* notably strengthened ability to withstand salinity and drought. The transgenic plants exhibited reduced membrane damage, lower ion leakage, and diminished ROS accumulation. Notably, *EcCaM* modulated key components of the abscisic acid (ABA) biosynthesis pathway (*RD29A*, *RD22*, *COR47*, *KIN*) and the salt overly sensitive (SOS) signaling cascade (*CBL4/SOS3*, *CIPK24/SOS2*, *NHX1/SOS1*, *CBL10*) (Jamra et al., 2021). These findings collectively highlight the translational potential of finger millet -derived genes in improving abiotic stress tolerance in major crops through molecular breeding and genetic engineering approaches.

**Table 4:** Genes and transcription factors conferring abiotic stress tolerance in finger millet and heterologous systems.

Gene(s)	Species (validation)	Functional role	Stress type	Key insights	Reference
*EcNAC67*)	Rice (*Oryza sativa*)	Transcription factor regulating stress-responsive genes	Drought, salinity	Improved biomass, water retention, and stress recovery	Rahman et al., 2016
*EcAPX1 (*Ecapx1*)*)	Finger millet (*Eleusine coracana*)	ROS detoxification (ascorbate peroxidase)	Drought	Higher expression linked to increased stress tolerance	Bhatt et al., 2013
*EcSOD, EcCAT1, EcGR, EcP5CS, EcCAX1, EcCAX3*)	Finger millet (*Eleusine coracana*)	Antioxidant defense, osmotic adjustment, ion homeostasis	Drought, salinity	Coordinated upregulation in tolerant genotypes	Rakkammal et al., 2023
*EcbZIP60*)	Tobacco (*Nicotiana tabacum*)	Transcriptional regulation of stress signaling	Drought	Improved photosynthesis and ER stress response	Babitha et al., 2015a
*EcbHLH57*)	Tobacco (*Nicotiana tabacum*)	Regulation of stress-inducible genes	Drought, salinity	Induced antioxidant and stress-responsive genes	Babitha et al., 2015b
*EcDehydrin7*)	Tobacco (*Nicotiana tabacum*)	Protein stabilization under dehydration	Drought	Improved osmotic adjustment and cell protection	Singh et al., 2015
*EcCaM*)	Arabidopsis (*Arabidopsis thaliana*)	Calcium signaling in stress response	Drought, salinity	Reduced ROS damage and improved membrane integrity	Jamra et al., 2021
*EcJAZ*)	Finger millet (*Eleusine coracana*)	Jasmonate signaling regulator	Multiple stresses	Regulates hormone signaling and stress adaptation	Sen et al., 2016
*EcGBF3*)	Arabidopsis (*Arabidopsis thaliana*), Tobacco (*Nicotiana tabacum*)	Transcription factor (ABA signaling)	Drought	Improved survival and water retention	Ramegowda et al., 2017
*EcNAC1*)	Tobacco (*Nicotiana tabacum*)	Multi-stress regulatory TF	Drought, salinity	Enhanced ROS scavenging and stress tolerance	Ramegowda et al., 2012
*EcbZIP17*)	Tobacco (*Nicotiana tabacum*)	ER stress and multi-stress response	Multiple stresses	Improved growth and stress recovery	Ramakrishna et al., 2018
*EcCIPK31-like*)	Setaria italica	Stress signaling (CBL–CIPK pathway)	Multiple stresses	Strong induction under drought and stress conditions	Nagarjuna et al., 2016
*EcDREB2A*)	Tobacco (*Nicotiana tabacum*)	Heat stress transcription factor	Heat	Improved survival under high temperature	Singh et al., 2021
*mtlD*)	Finger millet	Osmotic adjustment	Drought, salinity	Improved stress tolerance during early growth	Hema et al., 2014

## 6 Eco-friendly and other molecular strategies for abiotic stresses tolerance in finger millet

### 6.1 Plant growth-promoting bacteria

Soil microorganisms have long been employed to enhance crop productivity, playing essential roles in nutrient cycling, phytohormone modulation, disease suppression, soil structure improvement, and microbial-assisted leaching and bioaccumulation of inorganic elements (Khatoon et al., 2020). Among these, plant growth-promoting rhizobacteria (PGPR) have shown significant potential in supporting crop cultivation under abiotic stress conditions through the stimulation of plant growth, better nutrient uptake, and induction of defense mechanisms (Ojuederie et al., 2019). In finger millet, three drought-tolerant PGPR strains exhibited the capacity to generate 1-aminocyclopropane-1-carboxylate (ACC) deaminase were shown to alleviate drought stress through modulation of ethylene levels and antioxidant systems. The application of these PGPR strains reduced oxidative stress by elevating the activity of major antioxidant enzymes, including SOD, GPX, CAT, and GR, alongside increased accumulation of osmoprotectants (Proline) and phenolic compounds (Chandra et al., 2020). Specifically, the single inoculant *Variovorax paradoxus* (RAA3) and a consortium of four ACC deaminase-producing strains: *Ochrobactrum anthropi* (DPC9), *Pseudomonas palleroniana* (DPB13 & DPB16), and *Pseudomonas fluorescens* (DPB15) strains were effective in improving drought resilience in finger millet by enhancing chlorophyll content, antioxidant activity, and cellular osmolyte levels (Chandra et al., 2020). Additionally, fluorescent *Pseudomonas* strains (SPF-33, SPF-37, and SPF-5) isolated from saline environments were assessed for their ability to enhance salinity tolerance in salinity-sensitive finger millet seedlings (Mahadik and Kumudini, 2020). The finger millet seedlings were treated with three isolates of the *Pseudomonas* strain and subjected to various salt stress concentrations (0 to 350 mM NaCl). Under salinity levels up to 350 mM NaCl, strains SPF-37 and SPF-33 significantly improved germination rate, plant height, spikelet number, LRWC, proline, chlorophyll, flavonoids, and phenolic content. These enhancements were accompanied by a marked reduction in lipid peroxidation and hydrogen peroxide accumulation, indicating a strengthened antioxidant defense system. The findings underscore the potential of specific PGPR strains as bioinoculants for strengthening tolerance to abiotic stresses during finger millet cultivation.

### 6.2 Humic substance

HS represent a vital component of soil organic matter, arising from the oxidative decomposition of microbial, plant, and animal residues (Olivares et al., 2017; Canellas and Olivares, 2014). Among these, humic acid (HA) plays a key role in promoting plant growth, regulating carbon and nitrogen cycling, and maintaining soil structure stability (Rakkammal et al., 2024b). In addition to improving soil health, HA has been shown to enhance plant resilience under abiotic stress conditions by modulating metabolic pathways and improving physiological functions. Under salt stress, HA contributes to increased crop tolerance by enhancing water-use efficiency, stomatal conductance, and antioxidant enzyme activity (Saidimoradi et al., 2019). HA application improves the adaptive capacity of finger millet against salt stress through coordinated biochemical, physiological, and molecular responses (Rakkammal et al., 2024b). Specifically, HA-treated seedlings exhibited improved growth performance, elevated photosynthetic pigment content, higher leaf relative water content (LRWC), and significant upregulation of antioxidant enzyme genes. This molecular activation was accompanied by enhanced activity of CAT, SOD, GR, and APX, ultimately resulting in lower ROS accumulation and reduced oxidative damage (Rakkammal et al., 2024b).

### 6.3 Nanotechnology

Nanotechnology has emerged as a promising approach for application in agriculture and food, owing to the unique physicochemical properties of nanoparticles (NPs), including high surface area, tunable morphology, and enhanced reactivity (Thilakarathna and Raizada, 2015; Tipu et al., 2021). In the agricultural sector, metal- and metal oxide-based NPs such as iron oxide (Fe_3_O_4_-NPs), zinc oxide (ZnO-NPs), silicon oxide (SiO_2_-NPs), copper (Cu-NPs), selenium (Se-NPs), and titanium dioxide (TiO_2_-NPs) have garnered significant interest due to their potential to improve crop resilience and productivity. Under abiotic stress conditions, NPs can enhance plant growth by promoting nutrient uptake and reducing oxidative damage, partly through the modulation of ROS and associated stress biomarkers such as H_2_O_2_ and MDA (Al-Khayri et al., 2023). In finger millet, urea-doped calcium phosphate nanoparticles (CaP-U NPs) have been reported to alleviate drought stress by upregulating antioxidant enzyme activities (SOD, CAT, GPX, APX), improving photosynthetic efficiency, stomatal conductance, LRWC, carbohydrate accumulation, and nitrogen metabolism (Mishra et al., 2023). Additionally, the applications of Fe_3_O_4_-NPs and ZnO-NPs enhanced the transcriptional expression of zinc and iron transporters, including *EcZIP1*, *EcZTP29*, *EcFER1*, *EcIRT2*, and *EcYSL2*, thereby improving micronutrient uptake under stress (Chandra et al., 2021). Chitosan-based nanoparticles (Ch-NPs) have also demonstrated the ability to enhance growth, yield, and resistance to blast disease in finger millet (Sarma et al., 2023, 2021).

### 6.4 Transgenic approach

Transgenic approaches enable the simultaneous introduction of multiple genes that contribute to greater abiotic stress adaptability. The successful development of transgenic crops relies on the precise identification of functional candidate genes and the establishment of efficient regeneration, and transformation systems. Advances in plant genomics have significantly accelerated the discovery of genes associated with stress adaptation, yield enhancement, and nutritional improvement. These genomic tools are increasingly being applied to finger millet to identify agriculturally and nutritionally important genes for targeted manipulation. Notably, progress in finger millet transformation has been achieved through both *Agrobacterium tumefaciens*-mediated gene transfer and particle bombardment techniques, although efficiency remains genotype-dependent and requires further optimization (Antony Ceasar, 2011; Bhatt et al., 2011; Kothari et al., 2004). Among the available transformation approaches, *Agrobacterium tumefaciens*-mediated gene transfer is the most commonly utilized method for finger millet. However, genetic transformation studies in finger millet remain limited compared to well-studied cereals like rice, sorghum, and maize (Antony Ceasar, 2011). To date, only a few reports have documented the successful enhancement of abiotic stress tolerance in finger millet through transgenic interventions.

One notable example involves the introduction of the *PcSrp* (*Porteresia coarctata Serine-rich protein*) gene, associated with a protein high in serine content isolated from salt-stressed roots of *Porteresia coarctata*, into finger millet via particle bombardment (Mahalakshmi et al., 2006). The resulting transgenic plants were able to complete seed development under high salinity, and their roots accumulated significantly higher levels of Na^+^ and K^+^ ions compared to non-transgenic controls under 250 mM NaCl stress (Mahalakshmi et al., 2006). Similarly, finger millet transformed with the bacterial *mtlD* gene (*mannitol-1-phosphate dehydrogenase*) through *Agrobacterium*-mediated delivery showed improved resilience to drought, salinity, and oxidative stress, exhibiting better growth than wild-type plants under stress (Hema et al., 2014). In another study, overexpression of the sorghum *SbVPPase* gene in another study conferred salt stress tolerance, evidenced by greater chlorophyll and proline levels, stronger antioxidant enzyme activities, and diminished MDA levels in transgenic finger millet lines (Anjaneyulu et al., 2014). Overexpressing the *AVP1* gene from *Arabidopsis thaliana* together with the *PgNHX1* gene from *Pennisetum glaucum* contributed to producing salt-tolerant finger millet plants with superior physiological performance in environments with elevated salt levels (Jayasudha et al., 2014). Despite these advancements, scaling these genetic improvements for agronomic use remains constrained by genotype-specific transformation efficiencies and a lack of reliable plant regeneration protocols. Future research should focus on identifying stress-responsive candidate genes and optimizing transformation systems across diverse finger millet cultivars. Such efforts will be pivotal in developing genetically engineered finger millet genotypes showing increased resilience to abiotic stress.

### 6.5 Genome editing technology

**Figure 4:**
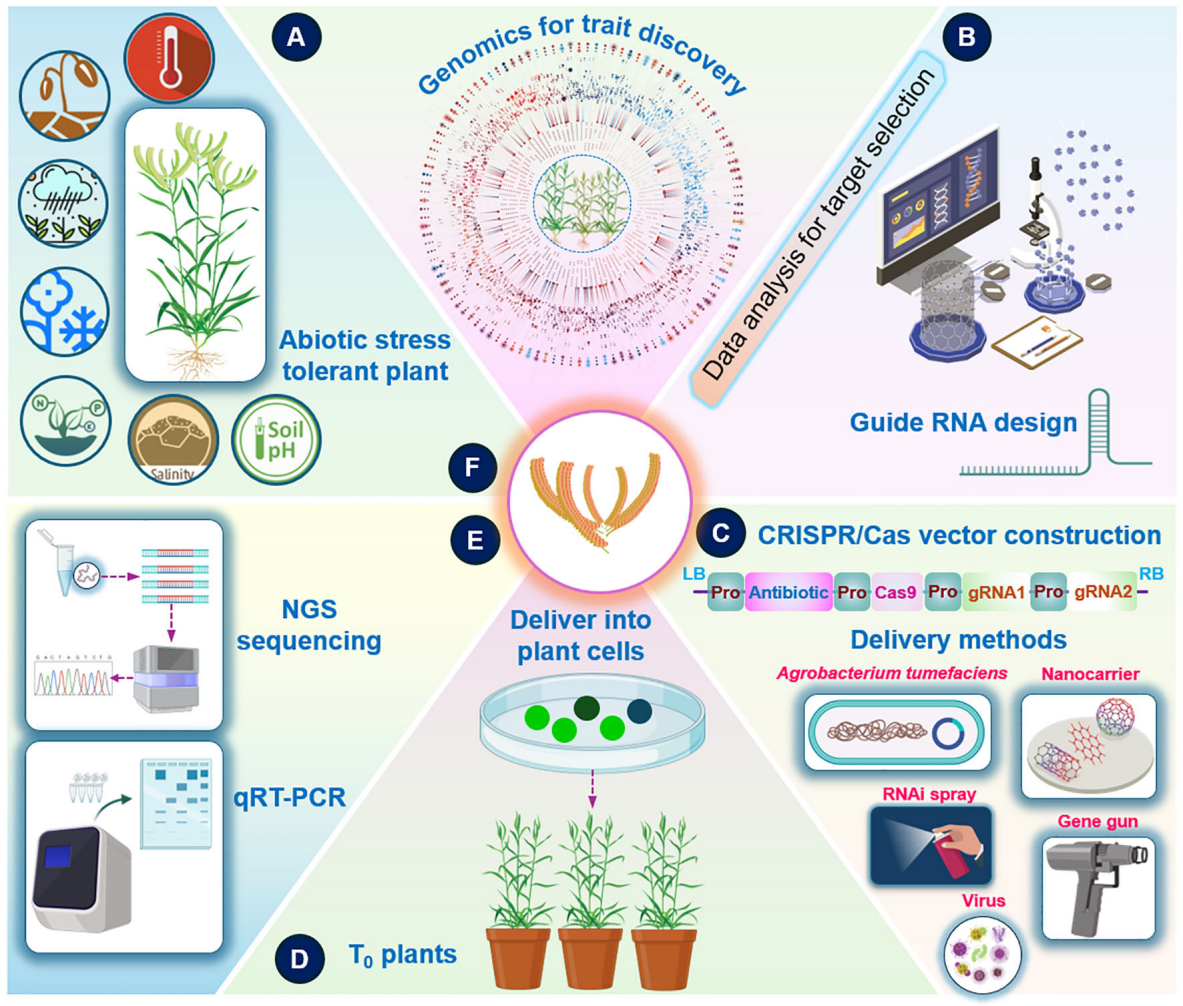
Stepwise CRISPR/Cas strategy to develop abiotic stress–tolerant finger millet. **(A)** Conceptual abiotic stress–tolerant finger millet ideotype used to define breeding and editing targets. Key climatic and soil constraints (drought, high and low temperature, waterlogging, salinity and suboptimal soil pH) that limit current productivity, and a representative plant illustrates the desired tolerant phenotype under these adverse conditions. **(B)** Genomics−driven trait discovery and target selection. Whole−genome and population−level sequence resources are analyzed using bioinformatic pipelines to identify loci, alleles and sequence motifs associated with abiotic stress tolerance that are suitable for genome editing, and to design corresponding guide RNAs. **(C)** CRISPR/Cas construct assembly and delivery options. A schematic gene−editing cassette shows promoter elements, Cas9, one or more guide RNA modules and selectable markers, alongside alternative delivery platforms including Agrobacterium tumefaciens–mediated transformation, nanocarriers, RNA or RNP sprays, viral vectors and particle bombardment (gene gun) for introducing the editing machinery into plant cells. **(D)** Regeneration and advancement of edited plants. Transformed cells are regenerated into T_0_ plants, which are grown in soil for initial phenotypic screening of edited lines under controlled conditions. **(E)** Molecular confirmation of edits. Candidate events are validated using next−generation sequencing to confirm targeted modifications and qRT−PCR to assess effects on transcript abundance of edited genes and downstream pathways. **(F)** Integration of genomic information, editing outcomes and phenotypic data across cycles of this pipeline enables rational stacking and fixation of favorable alleles, accelerating the development of finger millet cultivars with durable tolerance to multiple abiotic stresses.

Recent advances in genome editing, particularly the CRISPR/Cas (Clustered Regulatory Interspaced Short Palindromic Repeats/CRISPR-associated nuclease protein) systems have enabled precise and efficient targeting of specific genes or genomic loci for modification. The CRISPR/Cas genome editing system offers a powerful approach to elucidate the genetic basis of stress resilience by precisely modifying genes playing a role in abiotic stress adaptation. This technique enables the functional characterization of both positive and negative regulators of key traits, which is a key factor in boosting crop performance in unfavorable environments. Targeted gene knockouts or edits in structural genes, regulatory elements, promoter regions, or transcription factors can lead to altered protein function, thereby modulating stress-associated signaling pathways, metabolic networks, and physiological responses. These molecular modifications ultimately contribute to the development of stress-tolerant crop varieties with enhanced biochemical and metabolic plasticity (Mbinda and Mukami, 2021; Kumar et al., 2023). **Figure 4** depicts the mechanistic framework of CRISPR/Cas9-mediated genome editing in plants. The CRISPR/Cas9 system has been successfully employed in several cereal crops, including foxtail millet (Zhang et al., 2021; Cheng et al., 2021), rice (Wang et al., 2017; Dong et al., 2020), sorghum (Liu et al., 2019; Char et al., 2020), and wheat (Liang et al., 2017), all of which share phylogenetic proximity with finger millet. This technology has facilitated the development of genetically modified cultivars with enhanced tolerance to various abiotic stresses. In rice, for instance, several negative regulators of drought tolerance have been identified, including *OsDST* (*drought and salt tolerance protein 1*), *OsDIS1* (*drought-induced SINA protein 1*), and *OsSRFP1* (*ring finger protein 1*). Silencing these genes via CRISPR/Cas9 resulted in enhanced activity of antioxidant enzymes, reduced H_2_O_2_ accumulations, and improved drought resilience (Kumar et al., 2023; Fang and Xiong, 2015). In wheat, knock-out of the mutants of the *TaHAG1* gene generated through CRISPR/Cas9 exhibited improved salt tolerance. Furthermore, the targeted disruption of *OsLCT1* and *OsNramp5*, which encodes cadmium transporter proteins in rice, contributed to lowering cadmium accumulation in grain tissues (Songmei et al., 2019).

Despite the growing interest in genome editing, most millets have not achieved comparable success resulting from minimal genomic resources and underdeveloped transformation approaches (Ceasar, 2022). A fully annotated and functionally validated reference genome is essential for accurately identifying target loci and designing guide RNAs required for CRISPR/Cas-mediated editing. In finger millet, several additional challenges hinder the widespread adoption of this technology, particularly the lack of robust, genotype-independent *in vitro* regeneration and transformation protocols (Kudapa et al., 2023). Although efforts have been made to optimize tissue culture systems using various explants and gene delivery methods, the outcomes remain highly cultivar-dependent and have yet to yield consistent or reproducible results across genotypes (Satish et al., 2017; Ignacimuthu and Ceasar, 2012; Kothari et al., 2004). Therefore, the development of a universally applicable transformation platform, alongside a fully annotated genome, is imperative to harness the maximum capability of CRISPR/Cas9-driven genome editing in finger millet. Such advancements would fast-track the breeding of climate-resilient, stress-tolerant cultivars and contribute meaningfully to global food and nutritional security.

## 7 Conclusion and future perspectives

This review provides new insights into the functional, biochemical, and molecular framework of abiotic stress tolerance in finger millet, shedding light on the mechanisms that enable its adaptation to diverse climatic conditions. We synthesize recent advancements in understanding how finger millet deploys complex defense systems, including osmotic regulation, antioxidant networks, and gene regulatory circuits, to cope with drought, salinity, temperature extremes, and heavy metal stress. Despite notable progress, several critical gaps remain unresolved, particularly in linking molecular responses to phenotypic outcomes under field conditions. The release of a high-quality finger millet genome presents unprecedented opportunities to dissect stress-responsive genes, metabolites, and signaling pathways at finer resolution. While genomic, transcriptomic, and proteomic analyses have laid a strong foundation, further exploration in metabolomics, ionomics, and high-throughput phenomics is essential to fully characterize the plant’s stress adaptation strategies. A deeper integration of these multi-omics platforms will enhance our mechanistic understanding of abiotic stress responses and facilitate the identification of robust molecular markers for breeding. Moreover, genome editing and genomics-assisted breeding provide valuable avenues to accelerate the development of stress-resilient finger millet cultivars. Given the demonstrated cross-genera transferability of finger millet genes, these tools also hold potential for transferring key abiotic stress tolerance traits into major cereal crops. Such translational applications will be instrumental in addressing hidden hunger and safeguarding food security in climate-vulnerable regions.
