# Supplementary material for: Integrative insights into abiotic stress tolerance in finger millet (Eleusine coracana (L.) Gaertn.): linking physiological, biochemical, and molecular perspectives for developing climate-smart cereals
Source: Front Plant Sci. 2026 May 18;17:1815325. doi: 10.3389/fpls.2026.1815325 (PMC13222847; doi:10.3389/fpls.2026.1815325)
Supplement: Supplementary file 1 [file Table1.doc]

**Table S1. Nutritional values of finger millet and its comparison with other millets and major cereals.**

| **Proximate composition (per 100 g)** | **Finger millet** | **Sorghum** | **Foxtail millet** | **Pearl millet** | **Little Millet** | **Brown Rice** | **White Rice** | **Maize** | **Barley** | **Wheat** |
| --- | --- | --- | --- | --- | --- | --- | --- | --- | --- | --- |
| **Energy (kcal)** | **320.7±2.3** | **334.13±3.1** | **331.7±2.3** | **347.9±4.3** | **346.3±4.5** | **353.7±2.3** | **356.35±3.5** | **334.13±5.9** | **315.72±4.5** | **321.94±5.4** |
| **Moisture (g)** | **10.89±0.6** | **9.01±0.7** | **14.23±0.4** | **8.97±0.6** | **11.36±0.1** | **9.33±0.3** | **9.93±0.7** | **9.26±0.5** | **9.77±0.3** | **10.58±1.1** |
| **Carbohydrate(g)** | **66.82±0.7** | **67.68±1.03** | **66.19±1.1** | **61.78±0.8** | **65.55±1.2** | **74.80±0.8** | **78.24±1.07** | **64.77±1.5** | **61.29±0.7** | **64.72±1.7** |
| **Total dietary ﬁber (g)** | **11.18±1.1** | **10.22±0.4** | **6.39±0.6** | **11.49±0.6** | **7.72±0.9** | **4.43±0.5** | **2.81±0.4** | **12.24±0.9** | **15.64±0.6** | **11.23±0.7** |
| **Protein (g)** | **7.16±0.6** | **9.97±0.4** | **8.92±1.09** | **10.96±0.2** | **10.13±0.4** | **9.16±0.7** | **7.94±0.5** | **8.80±0.4** | **10.94±0.5** | **10.59±0.6** |
| **Fat (g)** | **1.92±0.1** | **1.73±0.3** | **2.55±0.1** | **5.43±0.6** | **3.89±0.3** | **1.24±0.08** | **0.52±0.05** | **3.77±0.4** | **1.30±0.2** | **1.47±0.05** |
| **Ash (g)** | **2.04±0.3** | **1.39±0.3** | **1.72±0.2** | **1.37±0.1** | **1.34±0.1** | **1.04±0.1** | **0.56±0.08** | **1.17±0.1** | **1.06±0.2** | **1.42±0.1** |
| **Minerals and trace elements (mg/100 g)** | **Finger millet** | **Sorghum** | **Foxtail millet** | **Pearl millet** | **Little Millet** | **Brown Rice** | **White Rice** | **Maize** | **Barley** | **Wheat** |
| **Calcium** | **364±58.0** | **27.60±3.7** | **15.27±1.2** | **27.35±2.1** | **16.06±1.5** | **10.93±1.7** | **7.49±1.2** | **8.91±0.6** | **28.64±3.4** | **36±5.6** |
| **Iron** | **4.62±0.3** | **3.95±0.9** | **2.34±0.4** | **6.42±1.04** | **1.26±0.4** | **1.02±0.3** | **0.65±0.1** | **2.49±0.3** | **1.56±0.1** | **3.97 ± 0.7** |
| **Magnesium** | **146±10.7** | **133±14.8** | **122±5.9** | **124±19.5** | **91.41±12.6** | **93.1±9.1** | **19.30±6.9** | **145±12.4** | **48.97±6.1** | **125±14.8** |
| **Manganese** | **3.19±0.8** | **1.19±0.1** | **0.33±0.05** | **1.12±0.1** | **0.23±0.08** | **1.70±0.4** | **0.73±0.2** | **0.71±0.08** | **1.24±0.1** | **3.19±0.5** |
| **Molybdenum** | **0.011±0.008** | **0.042±0.02** | **0.020±0.007** | **0.050±0.02** | **0.020±0.01** | **0.053±0.02** | **0.056±0.03** | **0.035±0.01** | **0.032±0.005** | **0.073±0.03** |
| **Phosphorus** | **210±58.4** | **274±35.7** | **101±5.2** | **289±25.3** | **130±27.5** | **267±64.9** | **96±16.30** | **279±35.3** | **178±26.8** | **315±41.8** |
| **Potassium** | **443±59.6** | **328±25.1** | **94±10.7** | **365±18.0** | **105±15.7** | **199±40.9** | **108±10.9** | **291±27.7** | **268±20.4** | **366±59.6** |
| **Sodium** | **4.75 ± 0.1** | **5.42 ± 0.2** | **3.35 ± 0.04** | **4.11±0.09** | **4.77 ± 0.1** | **3.64 ± 0.08** | **2.34 ± 0.2** | **4.44 ± 0.1** | **7.56 ± 1.5** | **2.50 ± 0.2** |
| **Zinc** | **2.53 ± 0.5** | **1.96 ± 0.3** | **1.65 ± 0.1** | **2.76 ± 0.3** | **1.82 ± 0.1** | **1.68 ± 0.3** | **1.21 ± 0.1** | **2.27 ± 0.2** | **1.50 ± 0.2** | **2.85 ± 0.6** |
| **Vitamins (per 100 g)** | **Finger millet** | **Sorghum** | **Foxtail millet** | **Pearl millet** | **Little Millet** | **Brown Rice** | **White Rice** | **Maize** | **Barley** | **Wheat** |
| **Thiamine (mg)** | **0.37±0.04** | **0.35±0.03** | **0.29±0.05** | **0.25±0.04** | **0.26±0.04** | **0.27±0.02** | **0.05±0.01** | **0.33±0.03** | **0.36±0.05** | **0.46±0.06** |
| **Riboﬂavin (mg)** | **0.17±0.008** | **0.14±0.01** | **0.20±0.01** | **0.20±0.03** | **0.05±0.008** | **0.06±0.01** | **0.05±0.006** | **0.09±0.009** | **0.18±0.03** | **0.15±0.04** |
| **Niacin (mg)** | **1.34±0.02** | **2.10±0.09** | **1.49±0.08** | **0.86±0.1** | **1.29±0.02** | **3.40±0.1** | **1.69±0.1** | **2.69±0.06** | **2.84±0.08** | **2.68±0.1** |
| **Pantothenic acid (mg)** | **0.29±0.19** | **0.27±0.02** | **0.63±0.07** | **0.50±0.05** | **0.60±0.07** | **0.61±0.04** | **0.57±0.05** | **0.34±0.03** | **0.14±0.02** | **1.08±0.2** |
| **Pyridoxine (mg)** | **0.05±0.007** | **0.28±0.02** | **0.07±0.01** | **0.27±0.009** | **0.04±0.005** | **0.37±0.03** | **0.12±0.01** | **0.34±0.01** | **0.31±0.02** | **0.26±0.03** |
| **Biotin (µg)** | **0.88±0.05** | **0.70±0.06** | **1.49±0.1** | **0.64±0.05** | **6.03±0.5** | **1.38±0.2** | **0.60±0.1** | **0.49±0.05** | **2.38±0.1** | **1.03±0.5** |
| **Folic acid (µg)** | **34.66±4.9** | **39.42±3.1** | **39.49±4.5** | **36.11±5.05** | **36.20±7.04** | **11.51±1.6** | **9.32±1.9** | **25.81±1.4** | **31.58±3.7** | **30.09±3.7** |

**Indian food composition Table 2017 (Longvah et al., 2017)**
